# Risk factors for mortality in hospitalized patients with COVID-19 at the start of the pandemic in Belgium: a retrospective cohort study

**DOI:** 10.1186/s12879-020-05605-3

**Published:** 2020-11-27

**Authors:** Karlijn van Halem, Robin Bruyndonckx, Jeroen van der Hilst, Janneke Cox, Paulien Driesen, Matthias Opsomer, Eveline Van Steenkiste, Björn Stessel, Jasperina Dubois, Peter Messiaen

**Affiliations:** 1 Department of Infectious Diseases and Immunity, Jessa Hospital, Stadsomvaart 11, 3500 Hasselt, Belgium; 2 Interuniversity Institute for Biostatistics and statistical Bioinformatics (I-BIOSTAT), Data Science Institute (DSI), Hasselt University, Hasselt, Belgium; 3 Laboratory of Medical Microbiology, Vaccine & Infectious Disease Institute (VAXINFECTIO), University of Antwerp, Universiteitsplein 1, 2610 Antwerp, Belgium; 4 Faculty of Medicine and Health Sciences, Hasselt University, Hasselt, Belgium; 5 Department of Intensive care Jessa Hospital, Hasselt, Belgium

**Keywords:** COVID-19, Coronavirus, Clinical characteristics, Mortality

## Abstract

**Background:**

Belgium was among the first countries in Europe with confirmed coronavirus disease 2019 (COVID-19) cases. Since the first diagnosis on February 3rd, the epidemic has quickly evolved, with Belgium at the crossroads of Europe, being one of the hardest hit countries. Although risk factors for severe disease in COVID-19 patients have been described in Chinese and United States (US) cohorts, good quality studies reporting on clinical characteristics, risk factors and outcome of European COVID-19 patients are still scarce.

**Methods:**

This study describes the clinical characteristics, complications and outcomes of 319 hospitalized COVID-19 patients, admitted to a tertiary care center at the start of the pandemic in Belgium, and aims to identify the main risk factors for in-hospital mortality in a European context using univariate and multivariate logistic regression analysis.

**Results:**

Most patients were male (60%), the median age was 74 (IQR 61–83) and 20% of patients were admitted to the intensive care unit, of whom 63% needed invasive mechanical ventilation. The overall case fatality rate was 25%. The best predictors of in-hospital mortality in multivariate analysis were older age, and renal insufficiency, higher lactate dehydrogenase and thrombocytopenia. Patients admitted early in the epidemic had a higher mortality compared to patients admitted later in the epidemic. In univariate analysis, patients with obesity did have an overall increased risk of death, while overweight on the other hand showed a trend towards lower mortality.

**Conclusions:**

Most patients hospitalized with COVID-19 during the first weeks of the epidemic in Belgium were admitted with severe disease and the overall case fatality rate was high. The identified risk factors for mortality are not easily amenable at short term, underscoring the lasting need of effective therapeutic and preventative measures.

**Supplementary Information:**

The online version contains supplementary material available at 10.1186/s12879-020-05605-3.

## Background

A major outbreak of a respiratory illness caused by the novel beta-coronavirus SARS-CoV-2 started in Wuhan, China late 2019 and has since spread rapidly throughout the world [1]. The disease, termed Coronavirus Disease 2019 (COVID-19), has been declared a pandemic by the World Health Organization (WHO), with over 37 million confirmed cases and one million deaths to date [2, 3].

Several publications addressed clinical characteristics of hospitalized COVID-19 patients during the early weeks of the pandemic and determined risk factors for severe disease in Chinese and US cohorts [4, 5]. However, there are differences between Chinese, US and European populations that could have an impact on the generalizability of these results and the translatability to European patients. Thus far comparable good-quality studies in a European context are scarce [6, 7].

Belgium was among the first countries in Europe with confirmed COVID-19 cases [8]. Since the first diagnosis on February 3rd, the epidemic has quickly evolved with Belgium, at the crossroads of Europe, being one of the hardest hit countries [9, 10]. The aim of our study was to describe the clinical characteristics of COVID-19 patients in a tertiary care centre early in the Belgian epidemic and identify independent risk factors for hospital mortality.

## Methods

This retrospective cohort study was conducted at the Jessa Hospital in Hasselt, a 981 bed non-academic tertiary care centre located in the centre of the outbreak in Belgium [11]. All patients aged 16 years or older, admitted to hospital for at least 24 h with confirmed COVID-19 until April 15, were included in the study.

Confirmed COVID-19 was defined as a positive real-time reverse transcriptase–polymerase chain reaction (RT-PCR) for severe acute respiratory syndrome coronavirus 2 (SARS-CoV-2) in a respiratory sample at any time during or before admission. Repeated testing was performed when there was a high clinical suspicion, but negative initial testing. Patients were treated in accordance with the Belgian guidelines in force at that time, which included hydroxychloroquine (400 mg twice daily for one day, followed by 200 mg twice daily up to day 5) when presenting with severe disease (defined as need for supplemental oxygen and chest X-ray abnormalities) [12]. The national guidelines recommended lopinavir / ritonavir as a second choice option, but this was not used in our center. Remdesivir was available only through a compassionate use program for patients admitted to the intensive care unit (ICU). Steroids as a systemic adjunctive treatment were not recommended and no specific advice was formulated yet regarding thromboprophylaxis or use of antibiotics. On March 31 we implemented intensified thromboprophylaxis in all patients admitted to the intensive care unit (ICU) [13]. The study was approved by the Ethics Committee of Jessa Hospital (ethical approval number 20.38-infect20.06). The requirement for informed consent was waived because of the retrospective nature of the study.

From the electronic medical records, we collected data on patient characteristics, comorbidities, and clinical symptoms, laboratory and radiology exams at hospital admission. Parameters were selected based on clinical relevance and previously published studies. Univariate logistic regression was used to assess significance of independent risk factors for in-hospital mortality. To reduce estimate’s bias because of small numbers of events per variable, a logistic regression model using Firth’s bias reduction method was used whenever cross table cell counts were below 5 [14]. To address that not all covariates were registered for all patients, missing values were imputed using multiple imputation (five imputations), using the fully conditional specification (FCS) method. This method of multiple imputation does not rely on the assumption of multivariate normality, instead using conditional distributions (regression models) specified for each variable with missing values. Each variable is therefore imputed conditional on the distribution of the remaining variables [15]. After imputation of missing values, a final multivariate logistic regression model was constructed through stepwise model building using pooled *p*-values (alpha 0.01) [14, 16]. Furthermore, we assessed how the comorbidity, age, lymphocyte count and lactate dehydrogenase score (CALL score), a previously published score for the prediction of progression to severe disease, performed for prediction of in-hospital mortality in our cohort, using the area under the receiver operating characteristic curve (AUC) [17]. Characteristics of patients admitted early in the epidemic (from first hospitalization until March 30), were compared with characteristics of patients admitted later (as from March 31) in the epidemic using univariate logistic regression. All analyses were conducted using SAS v9.4.

## Results

**Fig. 1:**
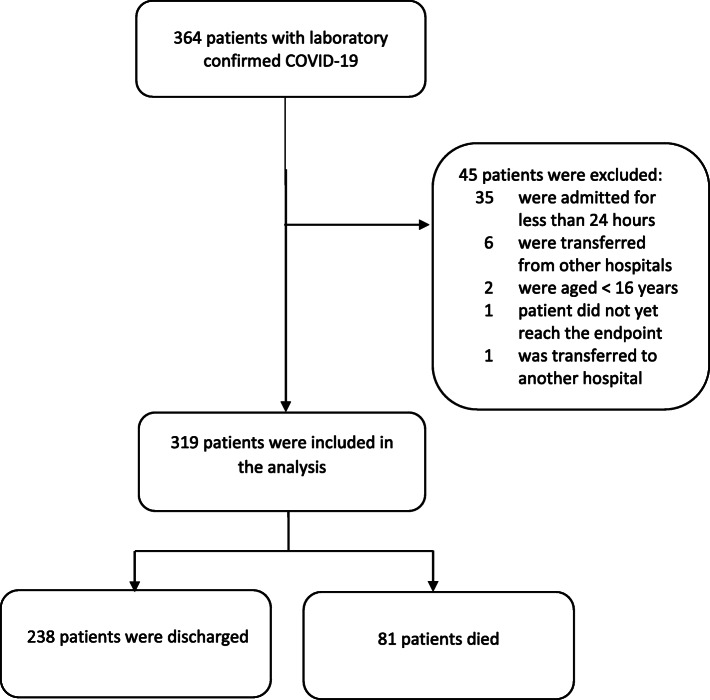
Study flow diagram

From when the first patient with COVID-19 was admitted on March 11 to April 15, 364 laboratory-confirmed COVID-19 patients had been admitted to our hospital, of which 319 patients met the inclusion criteria (Fig. 1).

Most patients were male (60%), the median age was 74 (IQR 61–83) years, with 70 patients (22%) being < 60 years of age. The most common comorbidities were hypertension (51%), coronary artery disease (23%), diabetes (20%), and chronic renal disease (20%) (Table 1). A significant proportion of patients (40%) was overweight (body mass index (BMI) 25–30) and 23% classified as obese (BMI > 30). Only 8% had known exposure to a confirmed COVID-19 case, in 7% nosocomial transmission was suspected and 3% of patients were healthcare personnel.

**Table 1:** Characteristics of patients at admission

	All patients (***n*** = 319)	Discharged (***n*** = 238)	Deceased (***n*** = 81)	p-value
**Baseline and demographic parameters**				
Median age (IQR) — yr	74 (61–83)	71 (59–79)	82 (76–86)	< 0.0001
Male — no. (%)	188 (58.93)	138 (57.98)	50 (61.73)	0.5530
Nursing home resident — no. (%)	30/318 (9.43)	17/137 (7.17)	13/81 (16.05)	0.0249
Smoking history				
- Current smoker — no. (%)	20/181 (11.05)	18/131 (13.74)	2/50 (4.00)	0.0619^c^
- Former smoker — no. (%)	63/181 (34.81)	42/131 (32.06)	21/50 (42.00)	0.2131
- Never smoker— no. (%)	98/181 (54.14)	71/131 (54.20)	27/50 (54.00)	0.9809
BMI (IQR)	26.75 (23.79–29.74)	26.35 (23.72–29.19)	28.17 (23.83–30.97)	0.0472
- Underweight^a^ - no. (%)	4/230 (1.74)	3/174 (1.72)	1/56 (1.79)	
- Normal weight^a^ - no. (%)	80/230 (34.78)	63/174 (36.21)	17/56 (30.36)	0.4206
- Overweight^a^ - no. (%)	92/230 (40.00)	73/174 (41.95)	19/56 (33.93)	0.2830
- Obesity^a^ — no. (%)	54/230 (23.48)	35/174 (20.11)	19/56 (33.93)	0.0390
- Not known	89	64	25	
Diabetes — no. (%)	65 (20.38)	38 (15.97)	27 (33.33)	0.0012
Hypertension — no. (%)	162 (50.78)	101 (42.44)	61 (75.31)	< 0.0001
- Use of ACE inhibitor	60/251 (23.90)	39/178 (21.91)	21/73 (28.77)	0.2530
- Use of ATII antagonist	30/250 (12.00)	18/177 (10.17)	12/73 (16.44)	0.1764
COPD — no. (%)	40 (12.54)	27 (11.34)	13 (16.05)	0.2805
Asthma — no. (%)	16 (5.02)	12 (5.04)	4 (4.94)	0.9286^c^
Coronary artery disease — no. (%)	72 (22.57)	47 (19.75)	25 (30.86)	0.0434
Chronic renal disease — no. (%)	62/318 (19.50)	34/237 (14.35)	28/81 (34.57)	0.0001
- eGFR 30–60	36/314 (11.46)	26/233 (11.16)	10/81 (12.35)	0.7743
- eGFR 15–30	17/314 (5.41)	5/233 (2.15)	12/81 (14.81)	< 0.0001
- eGFR < 15	3/314 (0.96)	0/233 (0.00)	3/81 (3.70)	0.0090^c^
- HD/PD	7 (2.19)	4 (1.68)	3 (3.70)	0.2582^c^
Cerebrovascular disease — no. (%)	48 (15.05)	30 (12.61)	18 (22.22)	0.0433
Immunocompromised — no. (%)	42 (13.17)	32 (13.45)	10 (12.35)	0.7992
- Immunosuppressive drugs	31 (9.72)	23 (9.66)	8 (9.88)	0.9556
- Active chemotherapy	16 (5.02)	10 (4.20)	6 (7.41)	0.2727
**Clinical symptoms**				
Mean duration of symptoms (IQR) – days	7 (3–10)	7 (4–10)	4 (1–7)	0.0007
- Not known	30	21	9	
History of fever^b^ — no. (%)	191 (59.87)	148 (62.18)	43 (53.09)	0.1510
Cough — no. (%)	211 (66.14)	161 (67.65)	50 (61.73)	0.3339
- purulent	47 (14.73)	36 (15.13)	11 (13.58)	0.7327
Dyspnea — no. (%)	208 (65.20)	152 (63.87)	56 (69.14)	0.3867
Thoracic pain — no. (%)	29 (9.09)	26 (10.92)	3 (3.70)	0.0488^c^
Myalgia — no. (%)	61 (19.12)	57 (23.95)	4 (4.94)	< 0.0001^c^
Diarrhea — no. (%)	49 (15.36)	38 (15.97)	11 (13.58)	0.6029
Abdominal pain— no. (%)	25 (7.84)	20 (8.40)	6 (6.17)	0.5088
Anorexia — no. (%)	93 (29.15)	76 (31.93)	17 (20.99)	0.0556
Nausea — no. (%)	55 (17.24)	45 (18.91)	10 (12.35)	0.1644
Vomiting — no. (%)	45 (14.11)	37 (15.55)	8 (9.88)	0.1908
Syncope — no. (%)	12 (3.76)	8 (3.36)	4 (4.94)	0.4583^c^
Headache — no. (%)	35 (10.97)	32 (13.45)	3 (3.70)	0.0117^c^
Confusion — no. (%)	29 (9.09)	20 (8.40)	9 (11.11)	0.4728
History of falling — no. (%)	19 (5.96)	8 (3.36)	11 (13.58)	0.0019
Anosmia — no. (%)	20 (6.27)	18 (7.56)	2 (2.47)	0.1103^c^
**Parameters**				
Temperature > 38 °C – no. (%)	52 (16.30)	41 (17.23)	11 (13.58)	0.4354
Respiratory rate (IQR) – breaths per min	16 (14–20)	16 (15–20)	16 (14–18)	0.5534
- Not measured	25	16	9	
Mean arterial blood pressure (IQR) - mmHg	94 (84–102)	93 (84–102)	97 (83–104)	0.6908
- Not measured	7	3	4	

^a^Body mass index (the weight in kilograms divided by the square of the height in meters) was categorized as: < 18.5, underweight; 18.5–25, normal weight; 25–30, overweight; > 30 obese

^b^Fever was defined as a measured body temperature > 38.0 °C

^c^Univariate logistic regression used Firth correction when small cell counts (< 5) occurred

The mean duration of symptoms at the time of presentation to the hospital was 7 days. Most patients had an important degree of hypoxemia, with 88% of patients having a partial pressure of oxygen (PaO2) below 80 mmHg without supplemental oxygen (Table 2). Twenty percent of all patients were admitted to the ICU, of whom 63% needed invasive mechanical ventilation. The most common complications were acute kidney injury, hyponatremia and acute respiratory distress syndrome (ARDS) (Table 3). The overall case fatality rate was 25%. More patients died in the wards (72%) compared to the ICU (28%).

**Table 2:** Laboratory and radiology findings at admission

	All patients (n = 319)	Discharged (n = 238)	Deceased (n = 81)	p-value
**Laboratory findings**				
White blood cell count (×10*9/L)	6.33 (4.89–8.50)	6.27 (4.80–8.41)	6.63 (5.16–8.97)	0.2258
- > 10	45/315 (14.29)	31/236 (13.14)	14/79 (17.72)	0.3505
- < 4	37/315 (11.75)	25/236 (10.59)	12/79 (15.19)	0.1795
- Not measured	4	2	2	
Lymphocyte count (×10*9/L)	0.75 (0.49–1.12)	0.81 (0.53–1.21)	0.63 (0.32–0.92)	0.0720
- < 1	211/314 (67.20)	150/236 (63.56)	61/78 (78.21)	0.0097
- Not measured	5	2	3	
Platelet count (×10*9/L)	191 (157–254)	196 (162–255)	171 (126–243)	0.0089
- < 150	71/316 (22.47)	42/237 (17.72)	29/79 (36.71)	0.0003
- Not measured	3	1	2	
C-reactive protein (mg/L)	76 (31–130)	68 (28–110)	96 (46–160)	0.0120
- Not measured	7	4	3	
Lactate dehydrogenase (U/L)	320 (250–420)	300 (230–400)	390 (280–475)	< 0.0001
- Not measured	42	33	9	
Ferritin (ug/L)	840 (390–1600)	735 (370–1500)	1200 (490–2100)	0.0073
- Not measured	90	62	28	
D-dimer (mg/L)	0.83 (0.53–1.41)	0.78 (0.51–1.28)	1.06 (0.62–1.67)	0.3039
- > 0.5 mg/L	185/237 (78.06)	133/177 (75.14)	52/60 (86.67)	0.1878
- Not measured	82	61	21	
Aspartate aminotransferase (U/L)	39 (28–56)	39 (26–54)	41 (31–60)	0.4091
- Not measured	12	7	5	
Alanine aminotransferase (U/L)	28 (20–43)	28 (20–43)	28 (20–43)	0.4939
- Not measured	13	8	5	
Gamma-glutamyl transferase (U/L)	45 (26–87)	46 (25–86)	42 (27–87)	0.5703
- Not measured	22	14	8	
Alkaline phosphatase (U/L)	68 (57–93)	67 (57–93)	70 (58–90)	0.4856
- Not measured	28	19	9	
Total bilirubin (mg/dl)	0.49 (0.38–0.68)	0.49 (0.38–0.67)	0.56 (0.38–0.72)	0.9666
- Not measured	24	15	9	
Creatinine (mg/dl)	0.99 (0.79–1.31)	0.94 (0.76–1.17)	1.24 (0.92–2.21)	< 0.0001
- Not measured	9	7	2	
eGFR (mL/min/1.73 m*2)^b^−	70 (48–87)	74 (58–91)	48 (25–71)	< 0.0001
- Not measured	10	8	2	
Sodium (mmol/L)	137 (135–140)	137 (134–139)	138 (135–141)	0.7515
- Not measured	7	5	2	
Potassium (mmol/L)	3.94 (3.60–4.26)	3.91 (3.60–4.26)	3.97 (3.58–4.26)	0.4519
- Not measured	7	5	2	
**Arterial blood gas**				
pH	7.48 (7.45–7.51)	7.48 (7.45–7.52)	7.47 (7.43–7.48)	0.0091
- Not measured	69	50	19	
pO2 without suppl oxygen (mmHg)	63 (55–71)	64 (56–73)	58 (50–67)	0.0190
- Hypoxemia^a^ — no. (%)	212/241 (87.97)	158/183 (86.34)	54/58 (93.10)	0.1105
- Not measured	78	55	23	
pCO2 (mmHg)	31 (28–34)	31 (28–35)	31 (28–34)	0.8152
- Not measured	78	55	23	
Lactate	1.50 (1.10–1.90)	1.40 (1.10–1.80)	1.60 (1.30–2.30)	0.0020
- Not measured	119	95	24	
**Radiology findings**				
Infiltrates on chest X-ray	205/292 (70.21)	154/220 (70.00)	51/72 (70.83)	0.8931
- Unilateral	64/292 (21.92)	49/220 (22.27)	15/72 (20.83)	0.7165
- Bilateral	140/292 (47.95)	104/220 (47.27)	36/72 (50.0)	0.6846

^a^Hypoxemia was defined as a PaO2 < 80 mmHg

^b^estimated glomerular filtration rate (eGFR) estimated using the CKD-EPI (Chronic Kidney Disease

Epidemiology Collaboration) equation

**Table 3:** Complications, Treatment, Outcome

	All patients (n = 319)	Discharged (n = 238)	Deceased (n = 81)	p-value
**Treatment**				
Hydroxychloroquine — no. (%)	164 (51.41)	130 (54.62)	34 (41.98)	0.0489
Antibiotics — no. (%)	227/318 (71.38)	161/237 (67.93)	66/81 (81.48)	0.0164
Systemic glucocorticoids — no. (%)	36/317 (11.36)	25/237 (10.55)	11/80 (13.75)	0.4436
Mechanical ventilation — no. (%)	71 (22.26)	46 (19.33)	25 (30.86)	0.0354
- Invasive ventilation	40 (12.54)	23 (9.66)	17 (20.99)	0.0112
- Non-invasive ventilation	22 (6.90)	15 (6.30)	7 (8.64)	0.4830
- High flow oxygen device	9 (2.82)	8 (3.36)	1 (1.23)	0.2779
**Complications**				
Acute respiratory distress syndrome — no. (%)	45 (14.11)	24 (10.08)	21 (25.93)	0.0008
Acute kidney injury^c^ — no. (%)	57 (17.87)	20 (8.40)	37 (45.68)	< 0.0001
- Need for renal replacement therapy	13 (4.08)	5 (2.10)	8 (9.88)	0.0049
Venous thrombo-embolism — no. (%)	25 (7.84)	20 (8.40)	5 (6.17)	0.5088
- Deep venous thrombosis	22 (6.90)	17 (7.14)	5 (6.17)	0.7634
- Pulmonary embolism	5 (1.57)	3 (1.26)	2 (2.47)	0.3853†
Hypoglycemia^a^ — no. (%)	16 (5.02)	9 (3.78)	7 (8.64)	0.1013
Hyperglycemia ^b^ — no. (%)	22 (6.90)	12 (5.04)	10 (12.35)	0.0343
Stroke — no. (%)	4 (1.25)	0 (0.00)	4 (4.94)	0.0021
Hyponatraemia < 135 mmol/L — no. (%)	157 (49.22)	120 (50.42)	37 (45.68)	0.4607
Hyponatraemia < 130 mmol/L — no. (%)	74 (23.20)	63 (26.47)	11 (13.58)	0.0133
**Outcome**				
Admission to the ICU — no. (%)	63 (19.75)	39 (16.39)	24 (29.36)	0.0123
Median length of stay in hospital — no. (%)	8 (5–14)	8 (5–14)	8 (5–12)	0.0273
- Not known	1	1	0	

^a^Hypoglycemia was defined as a serum glucose level of < 70 mg/dl

^b^Hyperglycemia was defined as a serum glucose level of > 250 mg/dl

^c^Acute kidney injury was defined according to the KDIGO guidelines as an increase in serum creatinine by 0.3 mg/dl within 48 h, or an increase in serum creatinin 1.5 times baseline creatinin, which is known or presumed to have occurred within the prior 7 days

**Fig. 2:**
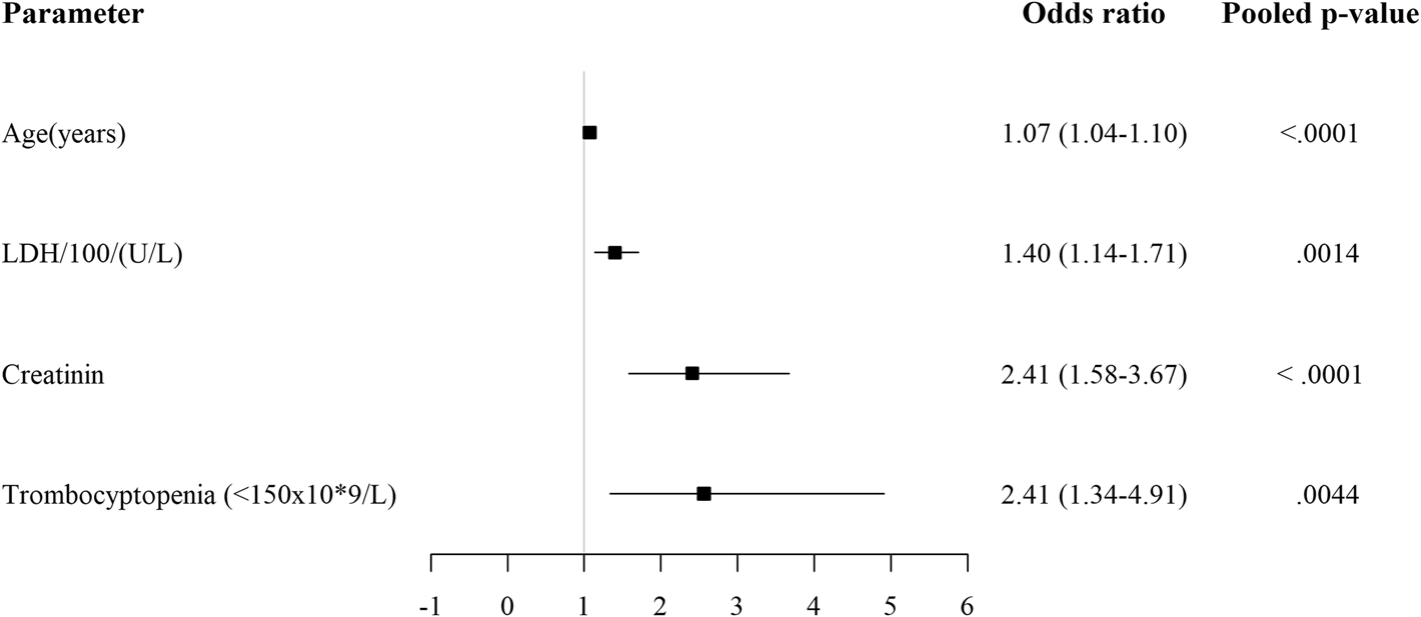
Admission early in the epidemic versus later in the epidemic

Univariate logistic regression showed that older age, residence in a nursing home, diabetes, pre-existing hypertension, chronic renal disease (eGFR < 30 ml/min), coronary artery disease and cerebrovascular disease were associated with a higher risk of death (Table 1). Smoking, either current or former, was not associated with a higher risk of death. Patients with obesity (BMI > 30) had an overall increased risk of death, but overweight on the other hand showed a trend towards lower mortality. Also, patients that died had reported a significantly shorter duration of symptoms at admission compared to patients that survived (4 vs 7 days, *p* = 0.0007). Lymphocytopenia, thrombocytopenia, high C-reactive protein (CRP), high lactate dehydrogenase (LDH) and high ferritin levels were significantly more present in patients who died of COVID-19 in hospital (Table 2), possibly reflecting a higher inflammatory state. Using multivariate logistic regression analysis we found the best predictors of in-hospital mortality to be older age, renal insufficiency, higher LDH levels and thrombocytopenia at admission (Fig. 2, Table S1 in supplementary materials).

We assessed the performance of the CALL score for prediction of in-hospital mortality using the area under the receiver operating characteristic curve (AUC) [17]. Over five imputed datasets, this score had an average AUC of 0.76 (min 0.75, max 0.76) which is considered excellent.

In patients admitted early in the epidemic (from first hospitalization until March 30), the mortality was significantly higher compared to patients admitted later (as from March 31) in the epidemic (Table 4). Mean age was not significantly different between these groups, but more patients admitted in the first weeks had hypertension. The proportion of patients admitted to the intensive care unit was not significantly different between groups.

**Table 4:** Admission early in the epidemic versus later in the epidemic

	Early ^a^ (***n*** = 206)	Late ^b^ (***n*** = 113)	p-value
Age — median (IQR)	74 (62–83)	73 (59–83)	0.3138
Hypertension — no. (%)	116 (56.31)	46 (40.71)	0.008
Diabetes — no. (%)	45 (21.84)	20 (17.70)	0.3754
Coronary heart disease — no. (%)	45 (21.84)	27 (23.89)	0.677
Chronic renal disease — no. (%)	42 (20.49)	20 (17.70)	0.5457
Number of days ill before admission – days (IQR)	6.5 (2–10)	7.8 (4–10)	0.0384
- Not measured	8	22	
Admission to ICU — no. (%)	44 (21.36)	19 (16.81)	0.3248
Death — no. (%)	62 (30.10)	19 (16.81)	0008

^a^Patients admitted between March 11 and March 30

^b^Patients admitted between March 31 and April 15

## Discussion

Our study describes the clinical characteristics, complications and outcomes of a large cohort of hospitalized COVID-19 patients in the early weeks of the pandemic in Belgium. In accordance with other reports, most patients were male [5, 7, 18]. The median age in our cohort was 74 (IGR 61–83), which is higher compared to recent studies in hospitalized patients from Italy (median 61, IQR 50–72), the US (median 63, IQR, 52–75) and China (median 41 years in 43 studies) [4, 5, 7]. The large majority of patients in our cohort presented with severe disease as defined by WHO [19], and we identified older age, renal insufficiency and higher LDH and thrombocytopenia as the most important risk factors for in-hospital mortality, which is in line with other reports [4, 7, 17, 20]. Because not all patient profiles were complete, these findings are based on the assumption that the missing data are missing at random, which means that conditional on the observed values included in the imputation model, the missing values are missing completely at random.

Comparing our results with previously published studies, some findings stand out. In our multivariate analysis, we did not find obesity to be amongst the most important risk factors for mortality. In univariate analysis, patients with obesity (BMI > 30) did have an overall increased risk of death, but overweight on the other hand showed a trend towards lower mortality. Several studies have described the increased need for mechanical ventilation and higher mortality in patients with obesity [20–23]. Several mechanisms are supposedly involved with this increased risk, mainly factors compromising respiratory physiology such as higher airway resistance, impaired gas exchange and lower lung volumes, but also increased risk of venous thromboembolism, a complication that is found to be very prevalent in patients with COVID-19 [24–26].

The association of overweight with lower mortality in both hospitalized and intensive care patients has previously been described and termed the obesity paradox [26, 27]. Despite the previously mentioned risk factors in obese patients, this was also found in a meta-analysis of observational studies in patients with pneumonia [27]. Whether this paradox represents a real protective effect of adipose tissue is uncertain and the topic of ongoing debate. In the setting of acute lung injury, it has been shown that patients with obesity have lower levels of proinflammatory cytokines, which could hypothetically be an advantage in COVID-19 [28]. Also, increased metabolic reserve is hypothesized to potentially be beneficial [27]. Because the obesity paradox thus far is based on observational studies only, it should be interpreted with caution and further data is needed to see whether this paradox truly exists and if so, applies to COVID-19 patients as well.

The incidence of venous thromboembolism in our cohort was low, which is in contrast with recent studies [24, 25]. This difference is most likely caused by detection bias, with a higher threshold for performing computed tomography (CT) scans due to a lower index of suspicion in the very beginning of the epidemic. No recommendations on thrombotic prophylaxis were included in the Belgian guidelines at the time. Currently, the increased risk of thromboembolic disease in COVID-19 is widely recognized, leading to better prophylactic strategies and aiding faster recognition and treatment, likely resulting in better outcome.

A previously published Chinese report found that in multivariate analysis comorbidity, older age, lymphocytopenia and high LDH at presentation were independent risk factors for COVID-19 progression to severe disease. From this, the “CALL” prediction score was developed to predict progression to severe disease [17]. However, most patients in our cohort already classified as having severe disease at admission. When assessing this score for prediction of hospital mortality, it also proved to perform well for the prediction of in-hospital mortality in our cohort.

There was no statistically significant difference in the administration of hydroxychloroquine between the deceased and discharged. In the beginning of the epidemic, off label chloroquine and/or hydroxychloroquine have both been widely advised in national guidelines as a possible treatment option for COVID-19, based mainly on in vitro data. Meanwhile several studies have shown no benefit of hydroxychloroquine on mortality [29–31]. Dexamethason and remdesivir, the only two therapies that have since shown to have an impact on outcomes in patients with severe COVID-19, were not advised at the time of our study [32, 33].

The overall case fatality rate in our cohort was high, similar to previously reported data from the UK and New York [5, 7], but higher compared to previous reports from China and Italy [4, 18]. This difference in reported case fatality rates in hospitalized patients is likely caused by a higher threshold for hospitalization compared to China and Italy, evident from the larger amount of patients being admitted with severe disease as defined by Wu et al. [34]. The fact that most patients died in the wards compared to the ICU reflects the attention for advanced care planning in our hospital consisting of a case-by-case assessment of the potential added value versus harm done by an intensive care admission, taking into careful consideration comorbidities, pre-admission performance status and patient wishes.

We found mortality to be higher in patients admitted in the first few weeks of the epidemic. Except for the prevalence of hypertension, the patient characteristics didn’t change however. We postulate that better understanding of the disease, its management and prevention of complications such as venous thromboembolism led to better treatment and overall outcome of patients admitted later in the epidemic.

Our study has certain limitations. The retrospective cohort analysis of a single centre may hamper generalization of the results in a broader geographical context, however our data are in line with other reported analyses. Furthermore, because not all patient profiles were complete, findings are based on the assumption that the missing data are missing at random, which means that conditional on the observed values included in the imputation model, the missing values are missing completely at random. However, whether missing data were truly missing at random or certain categories of patients were more frequently missing data is unknown. Also, we did not correct for multiple comparisons, making predictors with *p*-values close to 0.05 less likely to be true associations.

## Conclusion

Patients hospitalized with COVID-19 during the first weeks of the epidemic in Belgium were admitted with severe disease, with an overall case fatality rate of 25%. In multivariate analysis we identified older age, and renal insufficiency, higher lactate dehydrogenase and thrombocytopenia but not obesity as the most important risk factors for in-hospital mortality. Mortality was higher for patients admitted in the first few weeks of the epidemic, compared to patients admitted later in the epidemic, most likely reflecting a learning curve in case management. The previously described CALL score was validated as a useful clinical tool for the prediction of COVID-19 related mortality. The identified risk factors for mortality are not easily amenable at short term, underscoring the lasting need of effective therapeutic and preventative measures.

## Supplementary Information

**Additional file 1: Table S1.** Multivariate analysis of risk factors for death versus hospital discharge.

## Data Availability

The datasets used and/or analysed during the current study are available from the corresponding author on reasonable request.

## Abbreviations

COVID-19: Coronavirus disease 2019
US: United States
IQR: Interquartile range
SARS-CoV-2: Severe acute respiratory syndrome coronavirus 2
WHO: World health organisation
RT-PCR: real-time reverse transcriptase-polymerase chain reaction
ICU: Intensive care unit
FCS: Fully conditional specification
CALL score: Comorbidity, age, lymphocyte count and lactate hydrogenase score
AUC: Area under the curve
BMI: Body mass index
PaO2: Partial pressure of oxygen
ARDS: Acute respiratory distress syndrome
eGFR: Estimated glomerular filtration rate
CKD-EPI: Chronic Kidney Disease Epidemiology Collaboration
KDIGO: Kidney Disease: Improving Global Outcomes
CRP: C-reactive protein
LDH: Lactate dehydrogenase
CT: Computed tomography
