# Correction to: Risk factors for mortality in hospitalized patients with COVID-19 at the start of the pandemic in Belgium: a etrospective cohort study

**DOI:** 10.1186/s12879-020-05690-4

**Published:** 2020-12-14

**Authors:** Karlijn van Halem, Robin Bruyndonckx, Jeroen van der Hilst, Janneke Cox, Paulien Driesen, Matthias Opsomer, Eveline Van Steenkiste, Björn Stessel, Jasperina Dubois, Peter Messiaen

**Affiliations:** 1 Department of Infectious Diseases and Immunity, Jessa Hospital, Stadsomvaart 11, 3500 Hasselt, Belgium; 2 Interuniversity Institute for Biostatistics and statistical Bioinformatics (I-BIOSTAT), Data Science Institute (DSI), Hasselt University, Hasselt, Belgium; 3 Laboratory of Medical Microbiology, Vaccine & Infectious Disease Institute (VAXINFECTIO), University of Antwerp, Universiteitsplein 1, 2610 Antwerp, Belgium; 4 Faculty of Medicine and Health Sciences, Hasselt University, Hasselt, Belgium; 5 Department of Intensive Care Jessa Hospital, Hasselt, Belgium

**Correction to: BMC Infect Dis (2020) 20:897**

**https://doi.org/10.1186/s12879-020-05605-3**

Following publication of the original article [1], the authors identified an error in the caption of Fig 2. The correct caption is given below .

The incorrect caption is: "Admission early in the epidemic versus later in the epidemic".

The correct caption is: "Multivariate analysis of risk factors for death versus hospital discharge".

The original article [1] has been updated.
