# Supplementary material for: Risk factors for mortality in hospitalized patients with COVID-19 at the start of the pandemic in Belgium: a retrospective cohort study
Source: BMC Infect Dis. 2020 Nov 27;20:897. doi: 10.1186/s12879-020-05605-3 (PMC7691970; doi:10.1186/s12879-020-05605-3)
Supplement: Supplementary file 1 — Additional file 1: Table S1. Multivariate analysis of risk factors for death versus hospital discharge. [file 12879_2020_5605_MOESM1_ESM.docx]

**Table S1. Multivariate analysis of risk factors for death versus hospital discharge**

| **Parameter** | **Pooled Odds Ratio (95% confidence interval)** | **Pooled p-value** |
| --- | --- | --- |
| Age (years) | 1.07 (1.04-1.10) | <0.0001 |
| LDH/100 (U/L)* | 1.40 (1.14-1.71) | 0.0014 |
| Creatinin (mg/dl)* | 2.41 (1.58-3.67) | <0.0001 |
| Trombocytopenia (<150 x10*9/L)* | 2.56 (1.34-4.91) | 0.0044 |
| * measured at admission | | |
